# Genetic Characterization of the Co-Invasive Rodent Parasite *Heterakis spumosa* (Nematoda, Heterakidae)

**DOI:** 10.3390/ani14182674

**Published:** 2024-09-14

**Authors:** Srisupaph Poonlaphdecha, Alexis Ribas, Kittipong Chaisiri, Serge Morand, Abigail Hui En Chan, Urusa Thaenkham

**Affiliations:** 1 Parasitology Section, Department of Biology, Healthcare and Environment, Faculty of Pharmacy and Food Science, Institut de Recerca de la Biodiversitat (IRBio), University of Barcelona, 08028 Barcelona, Spain; spoonlaphdecha@ub.edu; 2 Department of Helminthology, Faculty of Tropical Medicine, Mahidol University, Bangkok 10400, Thailand; kittipong.cha@mahidol.ac.th (K.C.); abigail.cha@mahidol.edu (A.H.E.C.); urusa.tha@mahidol.edu (U.T.); 3 IRL HealthDEEP, CNRS—Kasetsart University—Mahidol University, Bangkok 10900, Thailand; serge.morand@umontpellier.fr; 4 Faculty of Veterinary Technology, Kasetsart University, Bangkok 10900, Thailand; 5 Department of Social and Environmental Medicine, Faculty of Tropical Medicine, Mahidol University, Bangkok 10900, Thailand

**Keywords:** *Heterakis spumosa*, Southeast Asia, rodent, mtCOI, ITS1

## Abstract

**Simple Summary:**

*Heterakis spumosa* Schneider, 1886 is a parasite commonly found in rodents, primarily associated with the black rat *Rattus rattus* and the brown rat *R. norvegicus*, two global invasive rodent species, and the house mouse *Mus musculus*. This parasite originated probably from Asia, and has been reported in various rodent species, but molecular information from its putative native region is still lacking. Our sampling efforts across Southeast Asia allowed us to collect and analyze this species from two localities in Lao PDR and five localities in Thailand. Additionally, specimens from Europe were included in the analysis. All specimens were analyzed using mtCOI gene and nuclear ribosomal ITS1. Our findings revealed the presence of two distinct clades of *H. spumosa*, with no discernible association with hosts and geographical localities.

**Abstract:**

*Heterakis spumosa*, a parasitic worm infecting rodents, is globally prevalent in black rats, brown rats, and house mice. It is hypothesized to originate from Asia due to its widespread presence in Southeast Asia in various Murinae. Previous molecular studies focused on European, African, and Japanese specimens, but none included samples from the putative native range. Rodents were collected between 2008 and 2015 across various localities in Southeast Asia and Europe, identified by morphology or genetic barcoding. Viscera were examined or preserved for later inspection. DNA was extracted from *H. spumosa*. PCR amplification targeting the mtCOI gene and ITS1 region was conducted in this study using newly designed primers (based on *Heterakis* reference sequences). PCR amplicons were subsequently sequenced and analyzed. In this study, the phylogenetic analysis using ITS1 sequences revealed that *Heterakis* samples from Thai and Laotian rodents belong to the species *H. spumosa*, exhibiting low genetic variation compared to samples from other regions. Genetic distance calculations using mtCOI sequences confirmed the marked distinction of *H. spumosa* from other *Heterakis* species. Our phylogenetic analyses using partial mtCOI and ITS1 sequences have significantly enhanced our comprehension of the genetic diversity and evolutionary history of the nematode *H. spumosa*.

## 1. Introduction

*Heterakis spumosa* Schneider, 1886, a parasitic worm infecting rodents, is globally prevalent in black rats *Rattus rattus* (Linnaeus, 1758), brown rats *R. norvegicus* (Berkenhout, 1769), and house mice *Mus musculus* (Linnaeus, 1758) [1,2,3,4,5,6,7]. The Asian origin of this nematode is hypothesized due to its widespread presence in Southeast Asia (SEA) in various Murinae from Cambodia, Lao PDR, and Malaysia. *Heterakis spumosa* is associated with the two globally invasive rodents, *R. rattus* and *R. norvegicus* [1,2]. Past molecular genetic studies on *H. spumosa* included the study by [3] on nuclear small subunit ribosomal DNA (18S rDNA) on worms from European rodents and by [4], who analyzed ITS-1, 5.8S, and ITS-2 in African and European specimens. A more recent study by [5] used mitochondrial cytochrome c oxidase subunit 1 (mtCOI) sequences in Japanese specimens. None of these studies included specimens from the putative native range.

The population structure of *H. gallinarum* (Schrank, 1788), a representative of the genus *Heterakis*, has been explored using a partial mtCOI gene [8] revealing an absence of genetic population structure across populations. As the host was the domestic chicken, determinants of the population structure were hypothesized to be related to the chicken trade, rather than natural factors that could affect wildlife populations, such as mountains in the case of rodents [9,10], or habitat fragmentation [11]. Therefore, this study aimed to explore the genetic diversity of *H. spumosa* for the first time, adding a new nematode studied from rodents in SEA. During extensive research on SEA rodent helminths, numerous nematodes belonging to *H. spumosa* were collected for molecular analysis.

## 2. Materials and Methods

### 2.1. Sampling

The collection of rodents was carried out between 2008 and 2015 (see methodological details in [12]). Rodents were identified by morphology or using species–specific primers and/or barcoding assignments (accessible in the ‘Barcoding Tool/RodentSEA’ section of the Community Ecology of Rodents and their Pathogens in Southeast Asia (CERoPath) project website [www.ceropath.org] (accessed on 3 June 2024). Viscera were examined in the field under a stereomicroscope or preserved in ethanol for subsequent inspection and in 70% ethanol according to standard protocols [12]. Concerning SEA localities, the individuals were from 2 localities in Lao PDR (Vientiane and Luang Prabang Provinces) and 5 from Thailand (Songkla, Chiang Rai, Nan, Loei, and Nongbua Lamphu Provinces). Localities outside the original range of *H. spumosa* were obtained from invasive rodents as follows: two *R. norvegicus* from the Province of Barcelona (Spain) and Kosice (Slovakia) and one from Antwerp (Belgium), as well as from the house mouse (*Mus musculus domesticus*) from the Province of Barcelona (Spain). Available sequences of *H. spumosa* in Genbank were also included (GenBank accession numbers JX845278, KF765409, LC626017, LC389877, MH571871). As an outgroup of the genus, we collected *Heterakis* from *Gallus gallus* (Linnaeus, 1758) collected in Udon Thani Province (Thailand), as well as *H. gallinarum* (GenBank accession number KP308348, KT310157, LC592777); *H. beramporia* Lane, 1914 (GenBank accession number: KU529971); *H. dispar* (Schrank, 1790) (GenBank accession number MF319969, NC042411, OM530143); *H. isolonche* Linstow, 1906 (GenBank accession number KM212953).

### 2.2. DNA Extraction

A single worm of each individual rodent was individually separated into 1.7 mL microcentrifuge tubes. The helminths were washed thoroughly with sterile distilled water to remove the ethanol. Total genomic DNA was isolated from each sample using the Geneaid genomic DNA mini kit (Geneaid Biotech Ltd., Taipei, Taiwan) following the manufacturer’s recommendations.

### 2.3. DNA Amplification and Sequencing

The genus-specific PCR primers were designed for both the mtCOI gene and nuclear ribosomal ITS1 region from reference sequences that were available in GenBank. The reference sequences used included species in genera *Heterakis*, *Syphacia*, *Apisculuris*, *Angiostrongylus*, and *Trichuris*. Briefly, reference sequences were aligned using ClustalX 2.1 and Bioedit 7.0 and primers were manually designed using the conserved region among *Heterakis* sequences [13,14]. The primer properties were then checked in OligoCalc for their GC content, primer melting temperature, and presence or absence of potential hairpin formation [15]. The information on the newly designed primers is in Table 1.

Amplification was conducted in a T100^TM^ thermocycler (Bio-Rad Laboratories, Hercules, CA, USA), with a final PCR volume of 50 µL. Each reaction contained 15 µL of 2x TopTaq master mix kit (Qiagen, Hilden, Germany), 0.1 µM of each primer, and around 1 ng/µL of DNA. The thermocycling profiles were: 94 °C for 3 min of initial denaturation; then 34 cycles of 94 °C for 30 s, 50 °C (Hs-COX1_Fwd/Rev), and 60 °C (ITS1-Heterakis-F/R) for 50 s, and 72 °C for 1 min; followed by a final extension of 72 °C for 5 min. Amplicons (386 bp for mtCOI gene and 563 bp for ITS1 region) were visualized on 1% agarose gel stained with SYBR^TM^ safe (Life Technologies, Carlsbad, CA, USA). PCR products were sequenced with a commercial company (Macrogen, Seoul, Republic of Korea) using the Sanger sequencing method with the same primers used for PCR amplification. For the ITS1 region, 8 representative sequences of *H. spumosa* and 1 sequence of *H. gallinarum* were sequenced, while for the COI gene, 36 representaive sequences of *H. spumosa* and 1 *H. gallinarum* were sequenced.

### 2.4. Sequence Alignment and Phylogenetic

The electropherograms obtained after sequencing were edited using Bioedit 7.0, and contiguous sequences were generated from forward and reverse electropherograms [13]. Subsequently, the sequences obtained were aligned with GenBank’s sequences using the ClustalX 2.1 program [14]. The aligned sequences underwent manual verification, and the resulting alignment was utilized to construct neighbor-joining (NJ) and maximum likelihood (ML) phylogenetic trees in MEGA 6.0 [16]. Using MEGA 6.0, parameters for NJ phylogenetic construction included a *p*-distance model and ML performed with the best-fit nucleotide substitution model with 1000 bootstrap iterations for tree topology support. The best-fit nucleotide substitution model for the ITS1 region was the Kimura 2 parameter model, while Tamura Nei with Gamma distribution was the best-fit for the *COI* gene. *Ascaris* and *Toxocara* were selected as outgroups to root the ITS1 phylogenetic tree, while *Ascaris*, *Toxocara*, and *Anisakis* were used for the mtCOI phylogenetic tree. The phylogenetic trees were visualized and labeled with FigTree 1.3.1 [17]. The sequences were deposited in GenBank (PQ151146-PQ151182) and (PQ152342-PQ15234259.

The partial mtCOI and ITS1 sequences obtained after sequencing and phylogenetic analysis were first used for species identification and subsequently utilized to examine genetic relationships among *H. spumosa* from various hosts. The hosts include *Bandicota indica* (Bechstein, 1799), *B. savilei* Thomas, 1916, *Berylmys bowersi* (Anderson, 1879), *B. berdmorei* (Blyth, 1851), *Mus musculus domesticus*, *M. pahari* Thomas, 1916, *Rattus norvegicus*, *R. tanezumi* (Temminck, 1844), *Sundamys muelleri* (Jentik, 1880) (Muridae family), and *Cannomys badius* (Hodgson, 1841) (Spalacidae family) from across different regions.

## 3. Results

**Figure 1:**
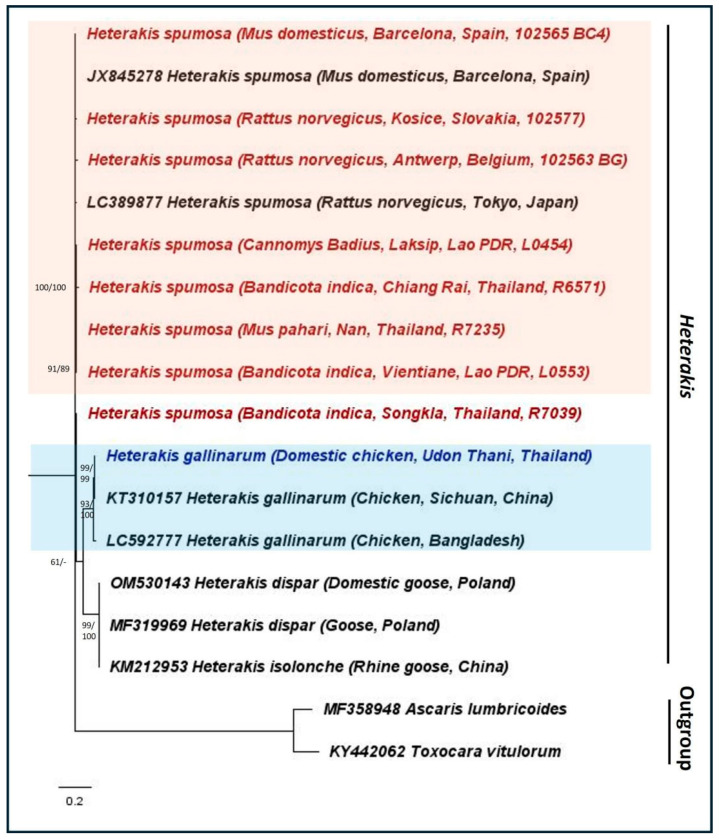
Maximum likelihood (Kimura 2 parameter model) phylogeny using the nuclear ITS1 region as the genetic marker. The numbers at the nodes indicate bootstrap support obtained through 1000 replications (ML/NJ). Only bootstrap values >50 are shown. Reference NCBI sequences are shown with their accession numbers. The representative sequences for *H. spumosa* and *H. gallinarum* are highlighted in ‘red’ and ‘blue’, respectively, with the host, location, and host ID given in parentheses.

The phylogenetic analysis using ITS1 sequences revealed that *Heterakis* samples from Thai and Laotian rodents belong to the species *H. spumosa* (Figure 1), exhibiting low genetic variation compared to samples from other regions. Our analyzed *H. spumosa* are genetically distant from *H. dispar*, *H. gallinanum*, and *H. isolonche*, which are parasites of birds.

**Figure 2:**
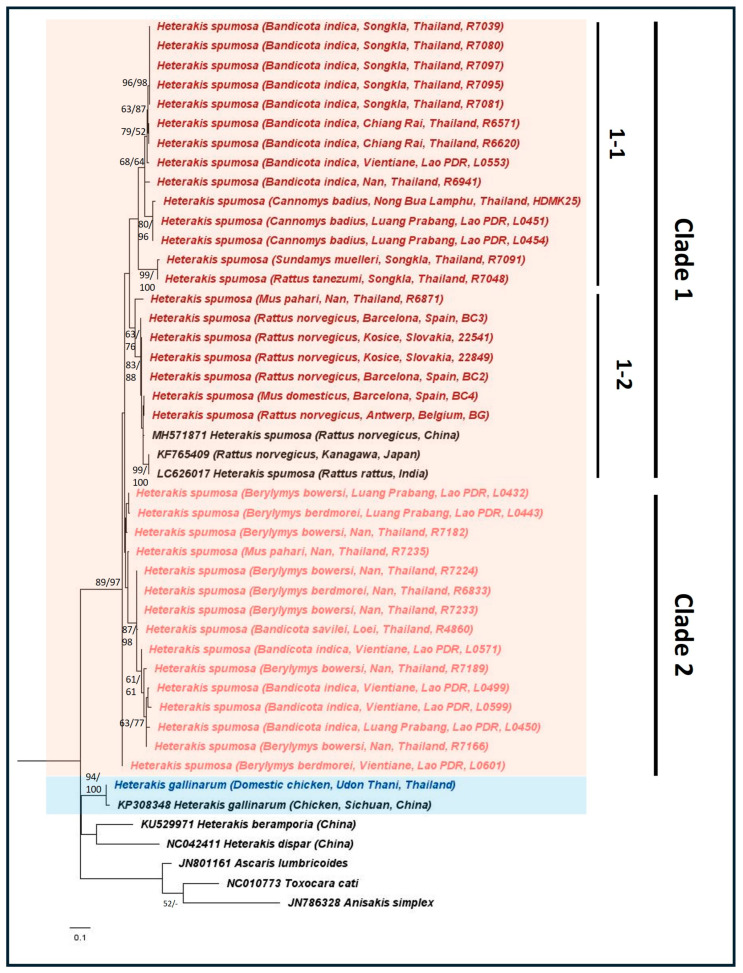
Maximum likelihood (Tamura Nei + Gamma distribution parameter model) phylogeny using the mtCOI gene as the genetic marker. The numbers at the nodes indicate bootstrap support obtained through 1000 replications (ML/NJ). Only bootstrap values >50 are shown. Reference NCBI sequences are shown with their accession numbers. The representative sequences for *H. spumosa* and *H. gallinarum* are highlighted in ‘red’ and ‘blue’, respectively, with the host, location, and host ID given in parentheses. The clade divisions of *H. spumosa* are labeled accordingly.

Using the mtCOI gene, phylogenetic analysis classified *H. spumosa* into two main clades: Clade 1, comprising a mixture from Europe, East Asia, and Southeast Asia, and Clade 2, exclusively from Southeast Asia (Figure 2). Genetic distance calculations using mtCOI sequences (Table 2) confirmed the marked distinction of *H. spumosa* from other *Heterakis* species, with 13 to 14% genetic distance obtained between *H. spumosa* and *H. dispar*. Additionally, the genetic distances between clades 1 and 2 were 7 to 8% lower than the distances obtained between *Heterakis* species. These results thus affirm that clades 1 and 2 are the same species (*H. spumosa*). Within our *H. spumosa* sequences, the genetic distance between clades 1 and 2 was 8.2%. Between both clades, a fixed nucleotide difference (G to A) was observed between them, while the other nucleotide differences were not specific for differentiation among the two clades. Despite the presence of nucleotide substitutions, they did not result in different amino acids being coded for.

## 4. Discussion

Our phylogenetic analyses using partial mtCOI and ITS1 sequences have significantly enhanced our comprehension of the genetic diversity and evolutionary history of the nematode *H. spumosa*. Notably, the findings from the mtCOI sequences, which revealed two primary clades, not only highlight the species’ genetic complexity but also prompt further investigation into the factors driving its diversification.

The identification of Clade 1, encompassing *H. spumosa* specimens from Europe, East Asia, and Southeast Asia, alongside Clade 2, exclusive to Southeast Asia, offers vital biogeographic insights. The intermingling of European and Asian lineages within Clade 1 suggests a shared evolutionary path, possibly indicating an Asian origin for *H. spumosa*. This aligns with the hypothesis of its transport by humans, mediated by *Rattus*, and the associated parasites from Asia to Europe [18]. Moreover, the *H. spumosa* exhibits significant intraspecific variation, even within the same locality or host, without a discernible pattern related to the host or location. Our results hint at a narrative where Clade 1 may signify the Asian origin of *H. spumosa*, which then spread to Europe. The observed genetic diversity in *H. spumosa* could result from its ability to infect and adapt to various rodent species, including the expansion of the distribution area in SEA of several murinae related to landscape changes that could put different genotypes of *H. spumosa* in contact.

Our results underscore the distinct genetic identity of *H. spumosa* compared to its nearest relatives, highlighting its unique evolutionary path. The notable intraspecific variation, evident even within the same localities or hosts, raises questions about the evolutionary processes involved. This variation could be linked to the species’ interactions with diverse rodent hosts, suggesting a scenario of host-driven diversification [19].

The absence of clear genetic patterns correlating with specific hosts or geographic locations is striking. This suggests that factors other than host species or geographic distribution might influence *H. spumosa*’s genetic structure. While our study is comprehensive, it has its limitations. The geographic scope covered, though broad, does not encompass the full distribution range of *H. spumosa*. This limitation may exclude unique genetic variations in unexplored populations, thus affecting our understanding of the species’ overall genetic diversity and biogeography. According to our results, the presumed original host of *H. spumosa* in Southeast Asia is not necessarily *R. rattus* or *R. norvegicus*. Other Murinae species could be the original host, with the parasite later spreading to other species or genera.

Additionally, relying solely on mitochondrial DNA markers like edited mtCOI sequences may not completely capture the total genetic diversity of *H. spumosa*. A more holistic approach, incorporating nuclear DNA markers or whole-genome analyses, would likely provide a richer understanding of its genetic landscape [20]. Moreover, the lack of detailed information on host–parasite interactions and the genetic diversity of the host species constrains our grasp of the co-evolutionary dynamics. This aspect is crucial for understanding the ecological and evolutionary complexities of *H. spumosa*.

The observed genetic diversity raises the possibility of cryptic species within what is currently classified as *H. spumosa* [21,22,23]. However, our study is limited by the absence of morphological or ecological data, and we cannot explore this hypothesis. The lack of environmental data also means the role of ecological factors in *H. spumosa*’s distribution and diversification remains unexplored. Furthermore, our study provides only a snapshot of the current genetic state, missing out on the temporal dynamics that could offer deeper evolutionary insights.

To overcome these limitations and enhance our understanding of *H. spumosa*, future research should expand geographic sampling, incorporate multi-locus genetic analyzes (including nuclear DNA and whole-genome studies), and conduct thorough investigations of host–parasite interactions while considering environmental factors. It is also imperative to explore the potential presence of cryptic species through morphological and ecological studies. Incorporating a temporal dimension, which includes historical samples, would shed light on the species’ evolutionary history.

This study contributes to the understanding of a parasite found in invasive populations of Norway and black rats worldwide. Comparing the molecular background of parasites from the original distribution area with those from newly colonized areas will help elucidate the co-invasion process.

## 5. Conclusions

In conclusion, while our study provides a better understanding of the genetic diversity of *H. spumosa*, a comprehensive and multi-faceted research approach is crucial for a more nuanced understanding of this species, its interactions with hosts, and its adaptability to various environments.

## Figures and Tables

**Table 1:** Primers designed for this study and the reference sequences for designing the primers.

Genetic Marker	Primers Designed	Annealing Temperature (°C)	PCR Amplicons Size (bp)	Accession No. of DNA Sequences Used for Primer Design
mtCOI	Hs-COX1_Fwd	50	386	MH571871, MH571870, NC_029838, NC_029839, OR177661, KY368775, KT900946, KT764937
	5′GAT TTT GCC TGC TTT TGG TAT 3′			
	Hs-COX1_REV			
	5′CTC ACC ACA TAA TAA GTA TCA 3′			
Nuclear rRNA ITS1	ITS1-Heterakis-F	60	563	JX845277, JX845278, MF319969, MH215352, EF464551, EF464554, EF464553, KP776414, KU575094
	5′GAG CCR CGT AGT GTA CTA CAA 3′			
	ITS1-Heterakis-R			
	5′TCG ACC CTC AGC CAG ACG TG 3′			

**Table 2:** Intraspecies genetic distances in *H. spumosa* and interspecies genetic distance between representatives of the genus *Heterakis* in birds.

Species/Genetic Distance		*H. spumosa*		*H. dispar*	*H. gallinarum*	*H. beramporia*
		Clade 1	Clade 2			
*H. spumosa*	Clade 1	7%				
	Clade 2	8.20%	4%			
*H. dispar*		14.30%	13.10%			
*H. gallinarum*		14.10%	13.30%	13.60%		
*H. beramporia*		14.00%	14.20%	14.40%	12.90%	

## Data Availability

All new data are available in the present publication.

## References

[1] Milazzo C., Goüy de Bellocq J., Cagnin M., Casanova J.C., Di Bella C., Feliu C., Fons R., Morand S., Santalla F. (2003). Helminths and ectoparasites of *Rattus rattus* and *Mus musculus* from Sicily, Italy. Comp. Parasitol..

[2] Milazzo C., Ribas A., Casanova J.C., Cagnin M., Geraci F., Di Bella C. (2010). Helminths of the brown rat (*Rattus norvegicus*) (Berkenhout, 1769) in the City of Palermo, Italy. Helminthologia.

[3] Zaleśny G., Hildebrand J., Popiołek M. (2010). Molecular Identification of *Heterakis spumosa* Schneider, 1866 (Nematoda: Ascaridida: Heterakidae) with comparative analysis of its occurrence in two mice species. Ann. Zool..

[4] Ribas A., de Bellocq J.G., Ros A., Ndiaye P.I., Miquel J. (2013). Morphometrical and genetic comparison of two nematode species: *H. spumosa* and *H. dahomensis* (Nematoda, Heterakidae). Acta Parasitol..

[5] Šnábel V., Utsuki D., Kato T., Sunaga F., Ooi H.K., Gambetta B., Taira K. (2014). Molecular identification of *Heterakis spumosa* obtained from brown rats (*Rattus norvegicus*) in Japan and its infectivity in experimental mice. Parasitol. Res..

[6] Julius R.S., Schwan E.V., Chimimba C.T. (2017). Helminth composition and prevalence of indigenous and invasive synanthropic murid rodents in urban areas of Gauteng Province, South Africa. J. Helminthol..

[7] Hancke D., Suarez O.V. (2018). Structure of parasite communities in urban environments: The case of helminths in synanthropic rodents. Folia Parasitol..

[8] Gu X., Zhu J.Y., Jian K.L., Wang B.J., Peng X.R., Yang G.Y., Wang T., Zhong Z.J., Peng K.Y. (2016). Absence of population genetic structure in *Heterakis gallinarum* of chicken from Sichuan, inferred from mitochondrial cytochrome C oxidase subunit I gene. Mitochondrial DNA.

[9] Wang Y., Feijó A., Cheng J., Xia L., Wen Z., Ge D., Sun J., Lu L., Li S., Yang Q. (2021). Ring Distribution patterns—Diversification or speciation? Comparative phylogeography of two small mammals in the mountains surrounding the Sichuan Basin. Mol. Ecol..

[10] Cuypers L.N., Sabuni C., Šumbera R., Aghová T., Lišková E., Leirs H., Baird S.J.E., Goüy de Bellocq J., Bryja J. (2022). Biogeographical importance of the Livingstone mountains in southern Tanzania: Comparative genetic structure of small non-volant Mammals. Front. Ecol. Evol..

[11] Brunke J., Radespiel U., Russo I., Bruford M., Goossens B. (2019). Messing about on the river: The role of geographic barriers in shaping the genetic structure of bornean small mammals in a fragmented landscape. Conserv. Genet..

[12] Ribas A., Chaisiri K., Morand S., Hugot J.P., Haukisalmi V., Henttonen H., Herbreteau V., Jittapalapong S., Rerkamnuaychoke W., Chaval Y., Cosson J.F., Morand S. (2011). Isolation of helminths from rodents. Protocols for Field and Laboratory Rodent Studies.

[13] Hall T.A. (1999). BioEdit: A User friendly biological sequence alignment editor and analysis program for Windows 95/98/NT. Nucleic Acids Symp. Ser..

[14] Thompson J.D., Gibson T.J., Higgins D.G. (2002). Multiple sequence alignment using ClustalW and ClustalX. Curr. Protoc. Bioinform..

[15] Kibbe W.A. (2007). OligoCalc: An online oligonucleotide properties calculator. Nucleic Acids Res..

[16] Tamura K., Stecher G., Peterson D., Filipski A., Kumar S. (2013). MEGA6: Molecular evolutionary genetics analysis Version 6.0. Mol. Biol. Evol..

[17] Rambaut A. (2009). FigTree.

[18] Morand S., Bordes F., Hsuan-Wien C., Julien C., Cosson J., Maxime G., Czirják G., Greenwood A., Latinne A., Michaux J. (2015). Global parasite and *Rattus* rodent invasions: The Consequences for rodent-borne diseases. Integr. Zool..

[19] Poulin R., Krasnov B.R., Morand S. (2006). Patterns of Host Specificity in parasites exploiting small mammals. Micromammals and Macroparasites: From Evolutionary Ecology to Management.

[20] Epps C.W., Keyghobadi N. (2015). Landscape genetics in a changing world: Disentangling historical and contemporary influences and inferring change. Mol. Ecol..

[21] Jueco N.L., Zabala Z.R. (1990). The nematodes of *Rattus norvegicus* and *Rattus rattus mindanensis*. Philipp. J. Vet. Med..

[22] Mohd Zain S.N., Behnke J.M., Lewis J.W. (2012). Helminth communities from two urban rat populations in Kuala Lumpur, Malaysia. Parasites Vectors.

[23] Dewi K., Purwaningsih E. (2013). Helminth parasites on rats in rubber plantation in Bogorejo Village, Gedongtataan Subdistrict, Pesawaran Regency, Lampung and their zoonotic review. Zoo Indones..

